# Transference of skills in robotic vs. laparoscopic simulation: a randomized controlled trial

**DOI:** 10.1186/s12893-021-01385-y

**Published:** 2021-10-28

**Authors:** John J. Kanitra, Nashwa Khogali-Jakary, Sahil B. Gambhir, Alan T. Davis, Michael Hollis, Caroline Moon, Rama Gupta, Pamela S. Haan, Cheryl Anderson, Deborah Collier, David Henry, Srinivas Kavuturu

**Affiliations:** 1 Department of Surgery, Ascension St. John Hospital, Detroit, MI 48236 USA; 2 Department of General Surgery, University of California, Irvine Medical Center, Orange, CA 92868 USA; 3 Department of Surgery, Michigan State University College of Human Medicine, 1200 E. Michigan Ave, Suite 655, Lansing, MI 48912 USA; 4 Montefiore Medical Center, Bronx, NY 10467 USA

**Keywords:** Laparoscopic skills, Laparoscopic training practice, Education, Training

## Abstract

**Background:**

Elucidating how robotic skills are best obtained will enable surgeons to best develop future robotic training programs. We perform a randomized controlled trial to assess the performance of robotic compared to laparoscopic surgery, transference of pre-existing skills between the two modalities, and to assess the learning curve between the two using novice medical students.

**Methods:**

Forty students were randomized into either Group A or B. Students practiced and were tested on a peg transfer task in either a laparoscopic simulator (LS) and robotic simulator (RS) in a pre-defined order. Performance, transference of skills and learning curve were assessed for each modality. Additionally, a fatigue questionnaire was issued.

**Results:**

There was no significant difference between overall laparoscopic scores (219 ± 19) and robotic scores (227 ± 23) (p = 0.065). Prior laparoscopic skills performed significantly better on robotic testing (236 ± 12) than without laparoscopic skills (216 ± 28) (p = 0.008). There was no significant difference in scores between students with prior robotic skills (223 ± 16) than without robotic skills (215 ± 22) (p = 0.162). Students reported no difference in fatigue between RS and LS. The learning curve plateaus at similar times between both modalities.

**Conclusion:**

Novice medical students with laparoscopic skills performed better on a RS test than students without laparoscopic training, suggesting a transference of skills from laparoscopic to robotic surgery. These results suggest laparoscopic training may be sufficient in general surgery residencies as the skills transfer to robotic if used post-residency.

## Background

In the era of decreased resident work hours and decreased autonomy our of concerns for patient safety, there is a need to augment the surgical apprenticeship model in surgical training [1]. With the introduction of new surgical technology, such as the robotic surgical system, there is a focus on the optimal method for teaching surgical skills [1]. The question remains whether performance of robotic assisted surgical procedures requires a different skill set compared to conventional laparoscopic surgery. Many hospitals have implemented the robotic surgical system; yet physicians, hospitals, and literature have yet to reach a consensus regarding its efficacy, training methods, and how it compares to standard laparoscopic surgery training [1–3]. As of 2016, general surgery residents demonstrated limited improvement in their robotic surgery skills during residency [2]. This was similarly seen with laparoscopic surgery leading to the Fundamentals of Laparoscopic Surgery (FLS) curriculum to formalize laparoscopic training using simulators [4–6]. At the present time, robotic surgery is not a requirement in general surgery residency, therefore, there is no formal surgical resident training curriculum analogous to FLS for robotics.

It has been suggested that there is a skills transference between laparoscopic and robotic assisted surgery [7, 8]. Conversely, others have demonstrated pre-existing laparoscopic skills lead to a worse performance in simple robotic tasks but an enhanced performance with difficult tasks [9]. Yet others suggest no or minimal transference of skills [10]. Understanding this would have clear implications on surgical training, in particular at programs that do not have access to robotic surgical systems or faculty to teach these skills.

There is extreme variability in the literature on the learning curve for laparoscopic and robotic surgery, but it appears that robotic surgery affords a faster learning curve than laparoscopic surgery for novice surgeons [11, 12]. Elucidating how robotic skills are best obtained will enable advancement in the development of future training programs.

We performed a randomized controlled trial (RCT) to assess the performance of novice medical students in robotic compared to laparoscopic surgery, transference of pre-existing skills between the two modalities, and to measure the learning curves. Additionally, we compared the mental and physical implications of the two modalities. With the current variability of the literature, we aim to add to the growing body of literature.

## Methods

**Fig. 1:**
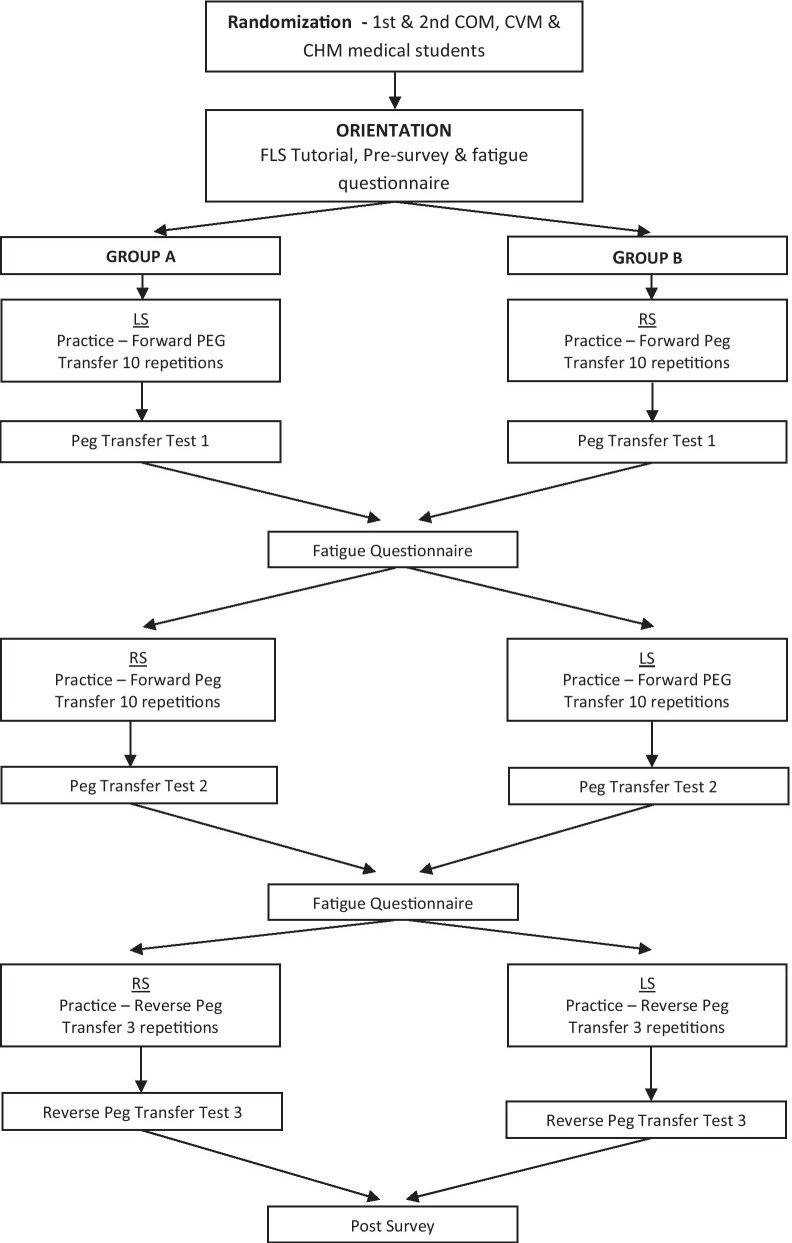
Laparoscopic and robotic simulation algorithm

All methods were performed in accordance with the relevant guidelines and regulation. Michigan State University institutional review board approved the study protocol (X13-1066). Novice medical students (no previous laparoscopic or robotic surgery experience) enrolled in the Michigan State University (MSU) College of Human Medicine (CHM), College of Osteopathic Medicine (COM) and College of Veterinary Medicine (CVM) were sent an email to request voluntary participation. Medical students were used to facilitate a larger powered study, which would have been considerably more difficult with residents and require multi-institution collaboration. The first 40 students to respond and met the inclusion criteria were informed of the study objectives and time commitment, obtained informed consent, advised there would be no compensation, then were subsequently enrolled in the study. The sample size for the study was based on our previous study looking at the influence of visual-spatial discordance in LS [13]. Each participant completed a pre-performance questionnaire to collect demographic information (age, sex, college) and if they describe themselves as expert video-gamers (1 = strongly disagree through 5 = strongly agree). Students were excluded if they reported any prior laparoscopic experience. Selected students viewed the FLS video tutorial introducing the peg transfer task and a standardized script was used to explain tasks and answer question. The students were randomized into either Group A or Group B using a random number generator. For the first round, Group A started practice and testing using LS while Group B started practice and testing using RS. For the second round, the groups switched so that Group A now practiced and was tested using RS and vice versa for Group B. For the final round, both groups remained in the same modality (RS or LS) they were in for round 2, but now practiced and were tested in the reverse alignment. Figure 1 outlines the algorithm used to test each group of participants.

Robotic and laparoscopic surgical skills were assessed using simulation models (RS = robotic simulation, LS = laparoscopic simulation). All practice and testing sessions were performed on a standard FLS training box and the da Vinci® robot (Intuitive Surgical, Inc., Sunnyvale, CA).

**Fig. 2:**
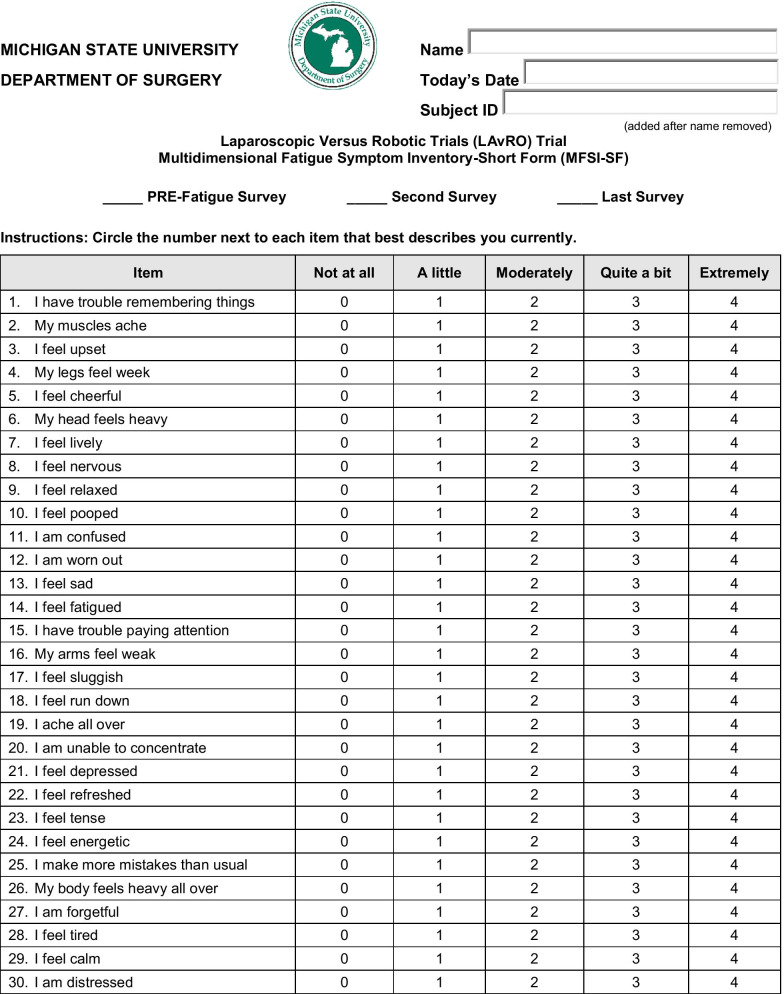
Multidimensional fatigue symptom inventory-short form. The fatigue symptom inventory/multidimensional fatigue symptom inventory © 1998 by H. Lee Moffitt Cancer Center and Research Institute, Inc is licensed under CC BY-NC-ND 4.0. To view a copy of this license, visit http://creativecommons.org/licenses/by-nc-nd/4.0/

Each participant completed a fatigue questionnaire after each test. For the fatigue questionnaire we used the Multidimensional Fatigue Symptom Inventory-Short Form (MFSI-SF) and scored according to their scoring scheme [14, 15]. The MFSI-SF is a 30-question assessment that measures general fatigue, physical fatigue, emotional fatigue, mental fatigue, and vigor fatigue (Fig. 2). Total score ranges from −24 to 96, with a higher score equating to more fatigue. The minimal clinically important difference ranges from 4.5 to 10.79 [16].

### Peg transfer exercise

The FLS curriculum peg transfer task was chosen to be used as the objective measurement as we felt it would be easiest for novice medical students to undertake. It consists of a square board with twelve pegs, separated evenly into two sections. Six beads are transferred with Maryland graspers from the first section of six pegs to the contralateral set of pegs utilizing a mid-air transfer between graspers, then back to the first section in the same fashion; this is considered one repetition. It has been shown that basic robotic skills are acquired quickly within 10 repetitions, prompting a 10-repetition parameter [17]. Students completed 10 repetitions in the forward alignment on both the FLS trainer box and the da Vinci. Three repetitions were performed in the reverse alignment. Given the rarity of reverse alignment in clinical practice, we chose to test students using only three repetitions to briefly assess performance. For ease of comparison, the FLS scoring system was used for both the FLS and robotic testing. FLS scores were calculated according to the formula: 300 – (seconds to complete transfer) – (10 × number of pegs dropped out of view and/or not transferred).

### Statistical analysis

Quantitative data are expressed as the mean ± standard deviation (SD), with the exception of the non-normally distributed data for testing in the reverse alignment, which are expressed as the median, followed by the range.

Comparisons of LS vs. RS were performed using the two-tailed unpaired t-test, with the exception of the testing in the reverse alignment, which was analyzed using the Mann–Whitney U test.

The transference of laparoscopic to robotic skills was analyzed by comparing Group A Test 2 (e.g. students with laparoscopic skills) and Group B Test 1 (e.g. students without laparoscopic skills). The transference of robotic to laparoscopic skills was analyzed by comparing Group A Test 1 (e.g. students without robotic skills) and Group B Test 2 (e.g. students with robotic skills). Laparoscopic and robotic performance were compared when performed in the reverse order (Group A, test 3 vs Group B, test 3).

To eliminate the confounder of comparing a different group of students with different duration of practice (Test 1 vs Test 2), we additionally performed a supplementary analysis looking at within-group analysis to compare Test 1 vs test 2 in Group A and the same analysis with Group B.

The fatigue questionnaire results were analyzed using mixed effects general linear modeling. In each analysis, the dependent variable was either the total MFSI-SF score, or a subscore (e.g., General Fatigue, Physical Fatigue). The independent variables were technique (LS vs. RS, reference: LS), timing (Group A vs. Group B; reference: Group A), sex (reference: Male), age (reference: Age < 25 years), and expert gamer (reference: No). Students were counted as being expert gamers if they indicated they agree or strongly agree with being an expert gamer in the pre-performance questionnaire. Significance was assessed at p < 0.05. All analyses were performed using Stata v.15.1 (StataCorp, College Station, TX).

## Results

Forty students participated in the study: n = 21 for Group A and n = 19 for Group B. Demographics for each group are found in Table 1.

**Table 1:** Group demographics

	Group A (%)	Group B (%)	*p*-value
College			0.509
MSU-COM	11/21 (52)	13/19 (68)	
MSU-CHM	8/21 (38)	6/19 (32)	
MSU-CVM	2/21 (10)	0/19 (0)	
Year			> 0.999
MS1	14/20 (70)	14/19 (74)	
MS2	5/20 (25)	5/19 (26)	
VS1	1/20 (5)	0/19 (0)	
Age ≥ 25 years	9/21 (43)	11/19 (58)	0.342
Females	11/21 (52)	8/19 (42)	0.516
Self-reported gaming ability			0.554
Expert	8/21 (38)	9/19 (47)	
Pre-test score^a^	−9 (−18, 30)	−8 (−20, 7)	0.545

MSU-COM, Michigan State University College of Osteopathic Medicine; MSU-CHM, Michigan State University College of Human Medicine; MSU-CVM, Michigan State University College of Veterinary Medicine; MS1, First-Year Medical Student; MS2, Second-Year Medical Student; VS1, First-Year Veterinary Medical Student

^a^Values represented by Median (Minimum, Maximum)

Multi-variate regression analysis comparing demographics to performance is found in Table 2. The only significant predictor of score was age (≥ 25 years old scored 10 points lower, p = 0.045).

**Table 2:** Multivariate regression analysis

	Reference variable	Coefficient^a^	95% CI^b^	*p*-value
Group B	A	−4.65	−14.3, 5.01	0.346
Female gender	Male	−0.88	−10.96, 9.20	0.864
Age > 25 years	< 25 years	−9.95	−19.70, −0.21	0.045
Robotic technique	FLS	7.93	−0.49, 16.34	0.065
Expert gamer^c^	No	3.11	−7.79, 13.05	0.621

MSU-COM, Michigan State University College of Osteopathic Medicine; MSU-CHM, Michigan State University College of Human Medicine; MSU-CVM, Michigan State University College of Veterinary Medicine

^a^Change in score compared to reference variable

^b^Confidence Interval

^c^Yes vs. No

There was no significant difference between overall LS scores (219 ± 19) and robotic scores (227 ± 23) (p = 0.065). Given there was not a statistically significant difference between LS and RS scores, a post-hoc power analysis indicates that 7 additional students (total n = 47) would be required to demonstrate a significant effect.

Students with prior laparoscopic skills performed significantly better on robotic testing (236 ± 12) than students without laparoscopic skills (216 ± 28) (p = 0.008). There was no significant difference in LS scores between students with prior robotic skills (223 ± 16) than without robotic skills (215 ± 22) (p = 0.162).

Within-group analysis for Group A demonstrated a significant improvement from Test 1 to Test 2 (215 ± 22 vs 236 ± 12; p < 0.001) whereas Group B did not demonstrate a significant difference (223 ± 16 vs 216 ± 28; p = 0.331). This suggests that when trained on LS first, robotic scores significantly improve but when trained on RS first, LS scores do not improve.

**Fig. 3:**
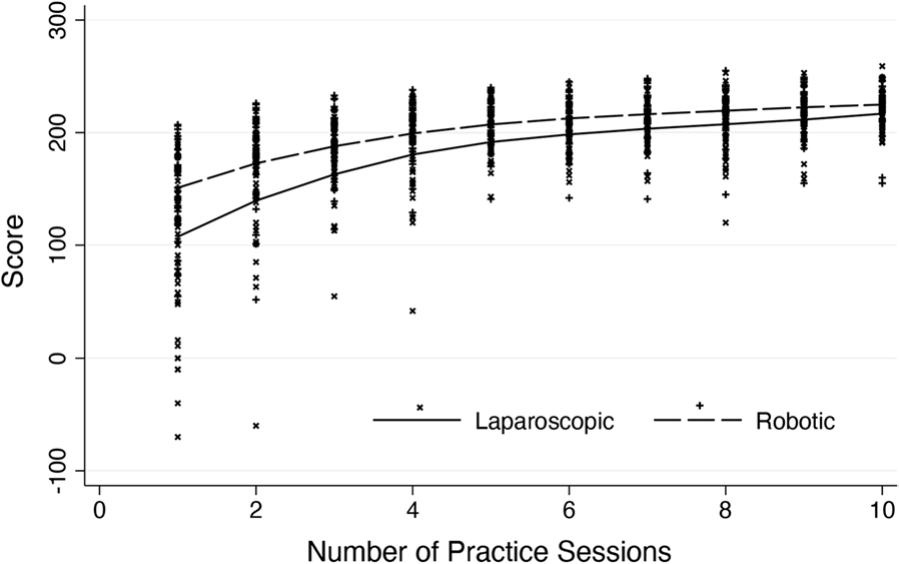
Learning curve for robotic and laparoscopic simulation

The learning curves of LS and RS demonstrate an early superiority of RS, with inflexion at round six for both modalities and a leveling off at similar scores at round 10, as seen in Fig. 3.

When tested in the reverse alignment, robotic performance (149, 56–199) was significantly better than laparoscopic performance (−80, −140–0) (p =  < 0.001).

Comparing LS vs RS fatigue questionnaire; general fatigue (2 vs 0, p = 0.067), physical fatigue (1 vs 0, p = 0.007), emotional fatigue (2 vs 0, p = 0.007), mental fatigue (1 vs 0, p = 0.007), and vigor fatigue (11 vs 13, p = 0.001). Total scores for the MFSI-SF for LS is -6 and for RS is -7 (p = 0.007), giving a difference of 1 point which does not meet the threshold of 4.5 to suggest a clinically important difference.

## Discussion

With the broadening spectrum of robotics, the question remains if a formal training program for general surgery residency is needed. The understanding of transference of skills between LS and RS, and learning curves between the different modalities could help answer this question. Our randomized control trial was primarily designed to address this.

We demonstrated that students who first underwent training in LS performed better in RS than students who started out in RS. To eliminate a potential confounder of one group having more practice than the other in our main comparison (i.e., Group A test 2 vs Group B test 1), we performed a within-group analysis for both Groups A and B, which confirmed the results of our main analysis. Adding validity to this study, when combined between all groups, overall LS scores compared to RS scores did not differ. The implications of this are important. At the present time, robotic training is not required in general surgery residency. Making robotics a requirement synonymous to FLS may not be feasible at all general surgery programs as they may lack robotic surgical systems, lack the robotic volume to train residents, or not have faculty to teach robotic skills. A transference in skills between LS and RS may give these programs some liberty in robotic training as laparoscopic training during surgical residency transfers to robotic if used post-residency. Even practicing on an FLS training box has been shown to improve robotic skills [8].

The results of our RCT alone should be interpreted with caution since only one task was performed, which is not representative of a comprehensive laparoscopic/robotic skills assessment. Baldonado et al. demonstrated that surgeons who have passed their video-assisted thoracoscopic (VATS) learning curve, did not demonstrate a definable learning curve in robotic surgery [18].

Two RCT’s with the same power (n = 40) and similar design to ours randomized subjects to either trained in laparoscopic or robotic simulation and were tested in the opposite modality [1, 10]. Hassan et al. demonstrated overall better scores with robotic surgery, however similar learning curves and a minimal transference of skill between both modalities [10]. Thomaier et al. demonstrated a transference of skills between both modalities, but to a lesser extent with robotic practice as opposed to laparoscopic practice [1]. The different conclusions regarding the transference of skills in the literature suggests more study is needed which justifies the current RCT. We demonstrated a transference of skill from laparoscopic to robotic but not the reciprocal. The learning curves were largely the same, with the same inflexion point and plateauing of scores, despite the initial higher score with robotics. Our RCT differed in that we also tested subjects in the reverse alignment and added the fatigue assessment.

Smaller trials also demonstrated a transference of skills from laparoscopic to robotic. Obek et al., n = 20, demonstrated a reciprocal transference of skills using intracorporeal knot tying, albeit subjects performed superiorly if trained on LS first [7]. Davila et al. n = 27, demonstrated no difference in robotic peg transfer or intracorporeal knot tying scores in subjects who received robotics training compared to no training, however subjects who had LS training performed better [8]. Panait et al., n = 28, demonstrating subjects with pre-existing laparoscopic skills performed inferiorly on simple tasks but superiorly on more complex tasks, suggesting a transference of skills from the laparoscopic platform to robotics which is enhanced in more complex tasks [9]. Finnerty et al., n = 36, demonstrated minimal improvement in robotic skills throughout surgical residency, however, laparoscopic experience correlated with superior robotic performance [2].

Additionally, we performed a test in the reverse alignment similar to our previous study to assess if the learning curve remains similar in different visual-spatial orientations [13]. As expected, RS performed significantly better likely owning to the ability of the robot to create a three-dimensional visualization.

With the focus of medical educators on resident burnout, the association of operative fatigue on overall resident fatigue becomes important. A 2019 meta-analysis on 10 papers suggested robotics to be ergonomically superior to laparoscopic surgery, however significant differences in study design of the literature limited the strength of their conclusion [19]. Laparoscopic surgery seems to invoke more muscle strain than robotic surgery, though interestingly this difference diminishes with increasing surgical expertise [20]. The current study did not demonstrate a difference in fatigue between both modalities, suggesting that adding RS to training curriculums does not have an impact on resident fatigue.

Limitations to this study include its relatively small power. However, our study size is the same or larger than others with a similar design. Additionally, we used novice medical students, which may have been the reason for our significant results as those less experienced in surgery will show a more significant improvement than experience surgeons [21]. The voluntary nature of this study could introduce a selection bias as it likely only enrolled medical students interested in surgery. The single institution design may affect the generalizability of the results. Given that only 10 sessions were performed in each modality, it is difficult to infer much from our learning curves besides that RS seems to easier to grasp early on for novice students. Finally, only one task (PEG transfer) was performed, which is not representative of a comprehensive skills assessment.

## Conclusion

Novice medical students with laparoscopic skills performed better on a RS test than students without laparoscopic training, suggesting a transference of skills from laparoscopic to robotic surgery. RS demonstrated an early superiority over LS, however the learning curves appear to plateau at similar times between both modalities. Additionally, we found no difference in self-reported fatigue between RS and LS. These results support existing literature suggesting that laparoscopic training may be sufficient in general surgery residencies as the skills transfer to robotic if used post-residency. The current studies add to the body of literature on the acquisition of robotic skills which is vital in surgical training programs that may not have access to robotic surgical systems.

## Data Availability

The datasets used and analyzed during the current study are available from the corresponding author on reasonable request.

## Abbreviations

CHM: College of Human Medicine
COM: College of Osteopathic Medicine
CVM: College of Veterinary Medicine
FLS: Fundamentals of Laparoscopic Surgery
LS: Laparoscopic simulation
MFSI-SF: Multidimensional Fatigue Symptom Inventory-Short Form
MSU: Michigan State University
RCT: Randomized Controlled Trial
RS: Robotic simulation
SD: Standard Deviation
